# Effect of Oral Intake of *Lactiplantibacillus plantarum* APsulloc 331261 (GTB1^TM^) on Diarrhea-Predominant Irritable Bowel Syndrome: A Randomized, Double-Blind, Placebo-Controlled Study

**DOI:** 10.3390/nu14102015

**Published:** 2022-05-11

**Authors:** Kyoungmi Jung, Areum Kim, Ji-Hae Lee, Donghyun Cho, Juyeon Seo, Eun Sung Jung, Hye-ji Kang, Jonghwa Roh, Wangi Kim

**Affiliations:** 1 Amorepacific Research and Innovation Center, Yongin-si 17074, Gyeonggi-do, Korea; areumkim@amorepacific.com (A.K.); seavet@amorepacific.com (J.-H.L.); cdh0529@amorepacific.com (D.C.); yeon8687@amorepacific.com (J.S.); rohjh@amorepacific.com (J.R.); katemina@amorepacific.com (W.K.); 2 HEM PHARMA Inc., Suwon-si 16229, Gyeonggi-do, Korea; esjung@hempharma.bio (E.S.J.); hjkang@hempharma.bio (H.-j.K.); 3 Global Green Research Development Center, Handong Global University, Pohang-si 37554, Gyeongbuk, Korea

**Keywords:** *Lactiplantibacillus plantarum* APsulloc 331261 (GTB1), green tea, irritable bowel syndrome, diarrhea

## Abstract

Irritable bowel syndrome (IBS) causes intestinal discomfort, gut dysfunction, and poor quality of life. This randomized, double-blind placebo-controlled trial evaluated the efficacy of *Lactiplantibacillus* (*Lp*., formerly *Lactobacillus*) *plantarum* APsulloc 331261 (GTB1^TM^) from green tea leaves in participants with diarrhea-predominant irritable bowel syndrome (IBS-D). Twenty-seven participants meeting the Rome IV diagnostic criteria were randomized for GTB1 or placebo ingestion for four weeks and follow-up for two weeks. The efficacy endpoints included adequate global relief of symptoms, assessment of intestinal discomfort symptom severity and frequency, stool frequency, satisfaction, and fecal microbiome abundance. Of all participants, 94.4% and 62.5% reported global relief of symptoms in the GTB1 and placebo groups, respectively, with significant differences (*p* = 0.037). GTB1 significantly reduced the severity and frequency of abdominal pain, bloating, and feeling of incomplete evacuation. The frequencies of diarrhea were decreased −45.89% and −26.76% in the GTB1 and placebo groups, respectively (*p* = 0.045). Hence, GTB1 ingestion improved IBS-D patient quality of life. After four weeks treatment, the relative abundance of *Lactobacillus* was higher in the GTB1 than in the placebo group (*p* = 0.010). Our results showed that GTB1 enhanced intestinal discomfort symptoms, defecation consistency, quality of life, beneficial microbiota, and overall intestinal health.

## 1. Introduction

Irritable Bowel Syndrome (IBS) is the most commonly reported functional gastrointestinal disorder. It affects ~11.2% (95% CI = 9.8–12.8%) of the global population. IBS has a high prevalence rate (7.0–17.0%), especially in Southeast and South Asia [1]. The incidence of IBS in South Korea is within ~6.6–16.8% [2,3,4], with a high disease prevalence and poor quality of life resulting from complex, recurring symptoms, which are socioeconomically burdensome [5,6]. IBS is now diagnosed according to the new Rome IV criteria adopted in 2016 [7]. It is a functional bowel disorder characterized by onset of symptoms in the past six months or earlier and recurrent abdominal pain related to defecation or changes in bowel habits over the past three months [7,8]. Abdominal bloating/distention, feeling of incomplete evacuation, and presence or absence of stool mucus are common symptoms even though these are not included among the diagnostic criteria for IBS [9]. IBS is classified into four subtypes based on their predominant bowel habits, namely, IBS with diarrhea (IBS-D), IBS with constipation (IBS-C), IBS with alternating constipation and diarrhea (IBS-M), and unclassified [7]. IBS-D accounts for ~1/3 of all cases [4,10] and commonly occurs in China and Singapore [11,12]. Most cases in Korea are IBS-M followed by IBS-D [11,12,13]. IBS-D patients present with diarrhea, abdominal pain, unpredictable bowel patterns, increased stool frequency, defecation urgency, and abdominal gas and bloating [8,14]. These symptoms markedly influence quality of life. Nevertheless, IBS treatment is challenging as its etiology is complex and influenced by gender, age, diet, visceral microbiota, psychological stress, and patient perspective [4,7,8,14]. Despite extensive research, IBS pathophysiology remains poorly understood. However, certain IBS symptoms are the result of interaction among visceral hypersensitivity, altered mucosal immune function, perturbations in gut microbiota abundance and diversity, CNS dysregulation, and genetic factors [9,14]. Hence, therapeutic modalities for IBS have focused on the foregoing pathophysiological mechanisms. Gut microbiota help maintain intestinal epithelial homeostasis and could, therefore, play major roles in IBS pathogenesis [15]. Probiotics are living microorganisms that maintain epithelial homeostasis and are often used to treat IBS symptoms [15,16]. Several studies identified the mechanisms of probiotics in IBS treatment. Randomized, placebo-controlled studies on IBS patients have revealed that *Lactobacillus* and *Bifidobacterium* are beneficial in this condition [17,18,19,20,21].

*Lactiplantibacillus plantarum* APsulloc 331261 (GTB1) is isolated from green tea leaves. This probiotic strain has excellent physiological properties. In a simulated stomach-duodenum passage experiment, GTB1 showed higher survivability than *Lp. plantarum* ATCC 14917. GTB1 has high acid and bile salt tolerance and can, therefore, successfully colonize the small intestine. Therefore, this strain is a good candidate as a beneficial probiotic. GTB1 adhered to Caco-2/TC-7 cells more tightly than either *Lp. plantarum* ATCC 14917 or *Lp. plantarum* 299v [22]. Probiotics that survive the upper digestive tract must be able to adhere to eukaryotic cells in order to colonize the gastrointestinal system. The ability of probiotics to attach and stick to epithelial cells despite intestinal peristalsis ensures that they will be able to stimulate the immune system, regulate the gut microbiota, and successfully compete against microbial pathogens for epithelial colonization [23]. GTB1 has significant advantages over other *Lactobacillus* strains in terms of the aforementioned physiological properties. In addition, GTB1 has anti-inflammatory efficacy and can significantly alter gut microbiota structure and proportions [23].

Prior research identified the excellent physiological characteristics of GTB1 such as intestinal cell adhesion, anti-inflammatory efficacy, and modulation of gut microbiota [22,23]. Hence, GTB1 will possibly also have beneficial intestinal health effects. Nevertheless, these have not yet been validated in human clinical trials. The objectives of the current study, then, were to evaluate the effects of ingesting the probiotic *Lp. plantarum* APsulloc 331261 on abdominal discomfort, bowel habits, fecal microbiome, and quality of life in patients with IBS-D.

## 2. Materials and Methods

### 2.1. Supplement Preparation

*Lactiplantibacillus plantarum* APsulloc 331261 (KCCM11179P, GTB1^TM^) was isolated from green tea cultivated on an organic tea field (Dolsongi tea field, Jeju Island, South Korea) and supplied by Amorepacific (Seoul, South Korea). The stability characteristics of this probiotic were previously described [22]. The selected daily intake of GTB1^TM^ was 1.0 × 10^10^ colony forming units (CFU) and within the probiotic dosage recommended by the Ministry of Food and Drug Safety, as well as the range of 10^9^–10^10^ CFU/d recommended for IBS-D in a prior meta-analysis [24]. The daily dosage was two capsules at 5 × 10^9^ CFU GTB1^TM^/capsule. The inactive ingredients were maltodextrin, silicon dioxide, and magnesium stearate. Both the placebo and the GTB1^TM^ supplement had the same flavor and taste. All supplements were produced in HACCP-certified plants according to GMP.

### 2.2. Study Participants

The participants were men and women over the age of 19 who were diagnosed with IBS according to Rome IV criteria [7]. During the 14-d screening period, participants with Bristol stool form scale (BSFS) ≥ 25% were enrolled as subjects with diarrhea type IBS (IBS-D). The exclusion criteria were as follows: probiotic or prebiotic (dietary fiber or oligosaccharide) consumption within four weeks before the clinical trial; systemic antibiotic consumption for more than one week within four weeks prior to the clinical trial; diagnosis and treatment of chronic bowel disorders other than IBS, secondary constipation-causing diseases, severe liver dysfunction, renal dysfunction, alcoholism, cardiovascular diseases, or immune diseases within four weeks before the clinical trial; consumption of any dietary supplements, functional foods, or medicines that could have the same or similar effects as the probiotics evaluated in the present study; acute or chronic diseases including uncontrolled metabolic disease; pregnancy, breast-feeding, or planned pregnancy during the study period; participation in any other clinical trial; and patient unsuitability deemed by the investigator. Twenty-seven participants who met all inclusion and exclusion criteria were enrolled in the present study. Participants were not allowed to take any other probiotics or antibiotics and were advised to maintain their lifestyle including their usual dietary habits and physical activities.

### 2.3. Study Design and Ethics

The study was undertaken according to a double-blind, randomized, placebo-controlled design with a total of 27 participants randomized to either GTB1 or placebo was planned and was conducted between November 2020 and January 2022 at the Amorepacific R&I center (Yongin, South Korea). This study was conducted according to the applicable principles of Good Clinical Practice. The study protocol was approved by the Institutional Review Board of the AMOREPACIFIC (2020-1MB-N9R, approval date: 8 October 2020) and was fully explained to all participants, who gave their written informed consent before participation. The study protocol was registered at clinicaltrials.gov (https://www.clinicaltrials.gov/ct2/show/NCT05277428, accessed on 8 April 2022), NCT05277428.

### 2.4. Study Procedure

**Figure 1:**
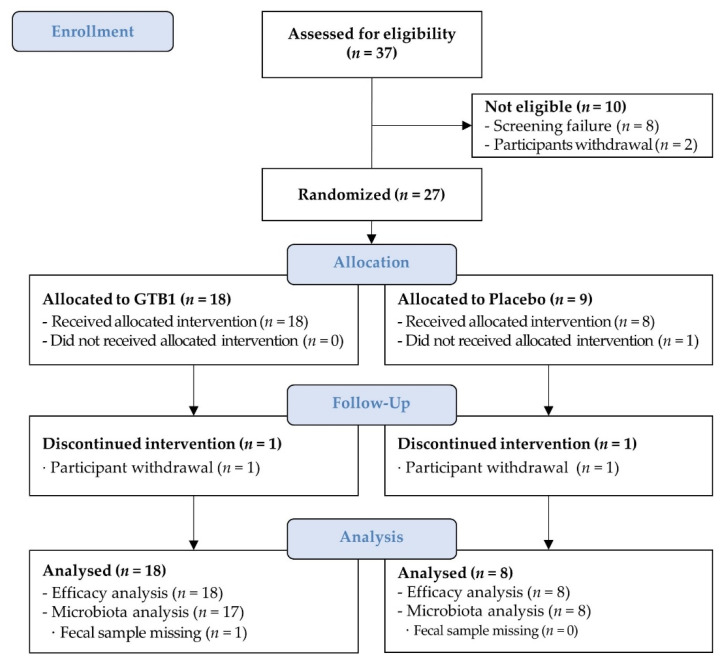
Participant flow chart.

A two-week screening period confirmed IBS symptoms and stool shape and frequency according to BSFS criteria. Of the 37 initial participants, eight did not meet other inclusion and exclusion criteria and two did not agree with the study. Thus, 27 participants who finally met all inclusion criteria were enrolled (Figure 1). Participants were randomly assigned to the GTB1 or placebo group in a 2:1 ratio. Group allocations were coded and concealed from the participants, laboratory technicians, and investigators throughout the trial. The randomized code was unblinded after all primary statistical analyses were performed. All participants consumed their assigned product with water once daily for four weeks. The efficacy endpoints were evaluated at baseline, and one, two, and four weeks after the initiation of supplementation and a two-week follow-up period. Vital signs were evaluated, physical measurements were made at all visits and adverse events were assessed during the intervention.

### 2.5. Evaluation of Efficacy Endpoints

The primary efficacy endpoint was bowel function improvement. Bowel function was assessed by (1) adequate global relief of IBS symptoms; (2) change in severity and frequency of intestinal discomfort symptoms, and; (3) change in frequency and type of defecation. Global relief of IBS was a dichotomous single item and participants were queried as follows: “Over the past week (seven days), have you had adequate relief of your IBS symptoms?” The answer was either YES or NO. The participants were then queried about the severity and frequency of their symptoms of intestinal discomfort including abdominal pain, abdominal bloating, and feeling of incomplete evacuation over the past seven days. Symptom severity was assessed using a visual analog scale (VAS; 0–10 points). Symptom frequency was assessed using a four-point scale ranging from one (none) to four (daily). Bowel movements were evaluated by requiring participants to write a defecation diary based on the seven-point ordinal BSFS scale [25,26] seven days before each visit. The average number of defecations per week and change in the frequency of diarrhea were evaluated using the defecation diary.

The secondary efficacy endpoint was the assessment of bowel movement satisfaction. Subjective bowel movement satisfaction was evaluated based on (1) bowel habit assessment; (2) improvement or worsening of IBS symptoms according to the Global Improvement Scale (GIS); and (3) overall quality of life assessment using the Patient’ Global Impression of Change (PGIC). The participants were asked about their bowel habits over the past seven days and evaluated according to a VAS ranging from 0 points (very dissatisfied) to 10 points (very satisfied). GIS was used to assess improvement or worsening of IBS symptoms on a seven-point Likert scale, namely, (1) substantially improved; (2) moderately improved; (3) slightly improved; (4) no change; (5) slightly worse; (6) moderately worse; and (7) substantially worse [27]. Supplemented participants whose GIS rating was “substantially improved” or “moderately improved” were treated as “improved”. Those whose GIS rating was “slightly improved,” “no change,” or “slightly worse” were treated as “non-response”. Those whose GIS rating was “moderately worse” or “substantially worse” were classified as “worsened” [28]. Participants were asked to rate their subjective satisfaction and change in their overall situation using the PGIC [29]. Participants were queried as follows: “Since the beginning of treatment in this clinical study, how would you describe any changes in activity limitation, symptoms, emotions, and overall quality of life associated with your painful condition?”. The answers were based on the following seven-point scale: (1) no change (or condition is worse); (2) almost the same (or hardly any change at all); (3) slightly better, but no noticeable change; (4) somewhat better, but the change has not made any real difference; (5) moderately better, and a slight but noticeable change; (6) better, and definite improvement that has made a real and worthwhile difference; and (7) a great deal better, and a significant improvement that has made all the difference.

### 2.6. DNA Extraction and Next-Generation Sequencing (NGS) of Fecal Microbiota

The analysis of fecal microbiota was performed in HEM PHARMA Inc. (Suwon, Korea). Stool samples were collected from patients at baseline, one, and four weeks after ingestion and were used to characterize the abundance and diversity of their fecal microbiota. Fecal bacterial genomic DNA was extracted with a Mag-Bind^®^ Universal Pathogen kit (Omega Bio-Tek, Norcross, GA, USA). Fecal samples were suspended in 275 μL SLX-Plus Buffer followed by bead milling in the MM400 mixer (Retsch, Haan, Nordrhein-Westfalen, Germany). Isolation, cleaning, and elution were conducted according to the manufacturer’s protocols. Ribosomal RNA gene amplicons for the Illumina Miseq System (Illumina, San Diego, CA, USA) describe a method for preparing samples for sequencing the variable V3-V4 regions of the 16S rRNA gene. The extracted fecal microbial DNA was amplified with 16S Amplicon PCR Forward Primer (5′-TCGTCGGCAGCGTCAGATGTGTATAAGAGACAGCCTACGGGNGGCWGCAG-3′) and 16S Amplicon PCR Reverse Primer (5′-GTCTCGTGGGCTCGGAGATGTGTATAAGAGACAGGACTACHVGGTATCT AATCC-3′). These amplicon primers, 2× KAPA HiFi HotStart ReadyMix (Roche, Basel, Basel-Stadt, Switzerland) and DNA were generated by PCR under conditions of 3 min at 95 °C, followed by 25 cycles at 95 °C for 30 s, annealing at 55 °C for 30 s, extension at 72 °C for 30 s and a final extension at 72 °C for 5 min. Subsequently sample DNAs were cleaned with HiAccuBead (AccuGene, Incheon, South Korea) and a magnetic stand. The Index PCR was performed by a using the IDT indexing primer (Integrated DNA technologies, Coralville, IA, USA) for Illumina Miseq System, 2× KAPA HiFi HotStart ReadyMix, and PCR grade water. PCR was carried out in a 95 °C for 3 min. 8 cycles of 95 °C for 30 s, 55 °C for 30 s, 72 °C for 30 s, then 72 °C for 5 min and held at 4 °C for PCR reaction. After clean-up step, the concentration of libraries was verified using the Qubit 4.0 (ThermoFisher Scientific, Waltham, MA, USA) with 1× dsDNA HS assay solution (ThermoFisher Scientific, Waltham, MA, USA) and sequenced using Illumina Miseq system. Reads were sorted using the unique barcodes for each PCR product. The barcode, linker and primer sequences were then removed from the original sequencing reads. The sequencing results was analyzed using Qiime2 bioinformatics pipeline and taxonomic assignment was performed with Silva 138 reference database (https://www.arb-silva.de/, accessed on 8 April 2022).

### 2.7. Statistical Analysis

Sample size was calculated using G*Power v.3.1.9.6 and was based on the changes in subject satisfaction reported by Philippe (2012) [17]. The authors used *Lp. plantarum*. The estimated power and significance were 80% and 5%, respectively. Based on the participant dropout rate, the minimum sample sizes required were 18 participants in the GTB1 group and nine participants in the placebo group. Statistical analyses were performed using JMP^®^ v.15.1.0 (SAS Institute Inc., Cary, NC, USA). Data were tested for normality using the Shapiro-Wilk test. Baseline measurements were assessed according to normality by two-sample *t*-test or Wilcoxon rank-sum test for continuous variables and the Chi-square test or Fisher’s exact test for categorical variables. Differences within the group from baseline were analyzed with a paired *t*-test or Wilcoxon signed-rank test for nonparametric data. Differences between the GTB1 and placebo groups were analyzed using a Chi-square test to analyze the global relief improvement rate, the frequency of intestinal discomfort symptoms, and the PGIC. Changes in the severity of intestinal discomfort symptoms, defecation frequency, quality of life and fecal microbiota levels were statistically evaluated using a two-sample *t*-test or Wilcoxon rank-sum test at each time point. Relative changes in the frequency of diarrhea were statistically evaluated using a two-sample *t*-test. Pearson’s correlation analysis was performed to determine the correlations between the changes in fecal microbiota and frequency of diarrhea. *p* < 0.05 indicated significant difference.

## 3. Results

### 3.1. Study Participants

The study participants (*N* = 27) were randomized at baseline and assigned either to the GTB1 group (*N* = 18; 1.0 × 10^10^ CFU GTB1^TM^/day) or the placebo group (*N* = 9). Twenty-seven participants completed the trial, of which 25 were included in the efficacy analysis. One withdrew consent and did not take the supplements while the other dropped out and missed sampling at four weeks. Both were excluded from the fecal analysis (Figure 1). There were no differences between the GTB1 and placebo groups in terms of age, gender, body mass index (BMI), vital signs, Rome IV criteria, intestinal discomfort, or defecation condition at baseline (Table 1). During the study period, temporary abdominal bloating, heartburn, and constipation were identified in certain subjects. However, there were no persistent or medication-requiring cases or serious adverse events.

### 3.2. Global Relief of IBS Symptoms

**Figure 2:**
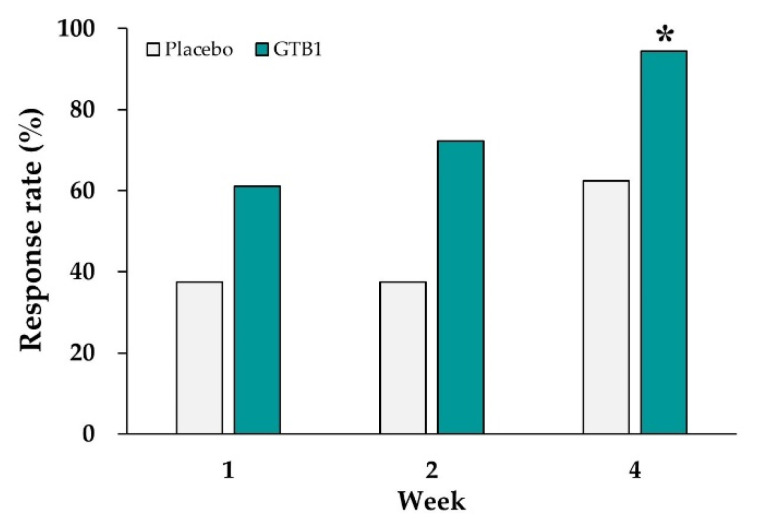
Response rates (%) for global relief of irritable bowel syndrome (IBS) symptoms after GTB1 and placebo ingestion. Data are presented as responder rates (%). * *p* < 0.05 as determined by Chi-square test.

In the GTB1 group, 61.1%, 72.2% and 94.4% of the respondents claimed adequate global relief of IBS symptoms after one, two, and four weeks ingestion, respectively, and the foregoing proportions increased with the duration of ingestion. At the end of the intervention, the response rates for adequate global relief of IBS symptoms were 94.4% in the GTB1 group and 62.5% in the placebo group. The difference in the proportion of respondents between groups was significant (*p* = 0.037, Figure 2).

### 3.3. Intestinal Discomfort Symptoms

**Figure 3:**
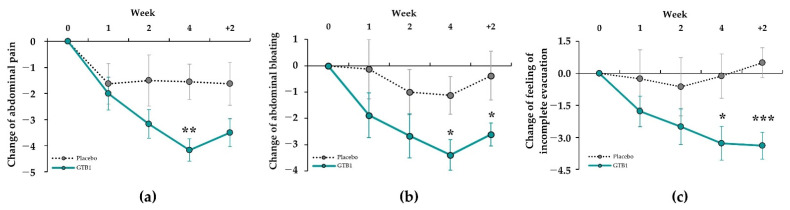
Change in severity of intestinal discomfort symptoms in GTB1 and placebo groups after one, two, and four weeks and follow-up week two. (**a**) Abdominal pain; (**b**) abdominal bloating; (**c**) feeling of incomplete evacuation. Differences were compared between each follow-up time point and before baseline. Data are means ± SEM. * *p* < 0.05, ** *p* < 0.01, *** *p* < 0.001 as determined by Wilcoxon rank-sum test or by two-sample *t*-test.

Changes in the severity of intestinal discomfort symptoms are presented in Table 2. At baseline, intestinal discomfort symptoms did not significantly differ between groups. In terms of the severity of abdominal pain, abdominal bloating, and feeling of incomplete evacuation, the GTB1 group significantly differed from the baseline at all points after ingestion. For the GTB1 group, abdominal pain significantly decreased from 5.83 ± 1.58 at baseline to 1.67 ± 1.08 at week four (*p* = 0.002). For the placebo group, abdominal pain did not significantly change (5.25 ± 1.39 at baseline vs. 3.75 ± 2.60 at week four) (Figure 3a). Severity of abdominal bloating and feeling of incomplete evacuation decreased to −3.39 and −3.28, respectively, at week four in the GTB1 group and significantly differed from the placebo group (*p* = 0.032 and *p* = 0.031). Significant difference persisted at follow-up after two weeks (Figure 3b,c). A 50% reduction in intestinal discomfort symptoms relative to baseline was deemed significant improvement at week four after ingestion. For the GTB1 group, there were 88.89% and 72.22% reductions in abdominal pain and abdominal bloating, respectively. Hence, there were significant improvements compared with the placebo groups (37.50% and 25.00%, respectively; Figure S1). The GTB1 group also showed a 55.56% improvement in feeling of incomplete evacuation but did not significantly differ from the placebo group (25.00%).

**Figure 4:**
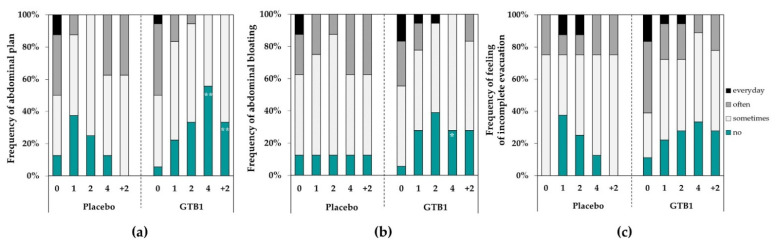
Frequency of intestinal discomfort symptoms during intervention period. (**a**) Abdominal pain; (**b**) abdominal bloating; (**c**) feeling of incomplete evacuation. Data for each visit are shown as % of all subjects. * *p* < 0.05, ** *p* < 0.01 as determined by Chi-square test.

In terms of frequency of intestinal discomfort symptoms, the GTB1 group improved compared to the placebo group. At baseline, the frequencies of abdominal pain in the GTB1 group were 5.6% and 94.4% for the subjects who answered ‘no’ and ‘yes (sometimes, often, daily)’, respectively, but improved to 55.6% and 44.4% after four weeks ingestion (*p* = 0.006, Figure 4a). The differences were significant compared with the placebo group and remained that way at follow-up week two (*p* = 0.003). Frequency of abdominal bloating was also significantly improved in the GTB1 group compared to the placebo group after four weeks (*p* = 0.017, Figure 4b). There were no significant differences between groups in terms of feeling of incomplete evacuation (Figure 4c).

### 3.4. Bowel Movement

**Figure 5:**
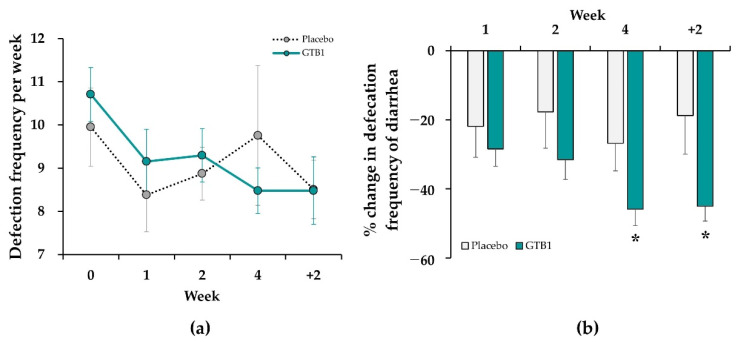
Changes in defecation frequency between GTB1 and placebo groups during intervention period. (**a**) Defecation frequency per week; (**b**) Relative mean change (%) in frequency of diarrhea (BSFS type 6–7). Data are means ± SEM. * *p* < 0.05 as determined by two-sample *t*-test.

Frequency of bowel movement per week did not significantly differ between the GTB1 and placebo groups at baseline. Frequency of bowel movement significantly decreased in the GTB1 group from 10.71 ± 0.62 at baseline to 8.48 ± 0.53 after four weeks (*p* < 0.001). In contrast, the placebo group exhibited no significant change in frequency of bowel movement during this time. Frequency of bowel movement in the GTB1 group decreased during the intervention, but did not significantly differ from that in the placebo group (Figure 5a).

At baseline in the GTB1 group, the stool consistencies were the watery type (BSFS 6–7) (69.07%), normal type (BSFS 3–4–5) (30.00%), and hard type (BSFS 1–2) (0.93%). At four weeks after ingestion, the proportions of the watery, normal, and hard stool consistency types were 23.19%, 76.10%, and 0.72%, respectively (Figure S2). For the placebo group, the proportions of the watery stool type were 63.22% at baseline and 36.46% after ingestion. The relative mean changes in frequency of diarrhea for the GTB1 and placebo groups were −45.89% and −26.76%, respectively, at week four (*p* = 0.045) and −45.09% and −18.79%, respectively, at follow-up week two (*p* = 0.014, Figure 5b).

### 3.5. Satisfaction Assessment

**Figure 6:**
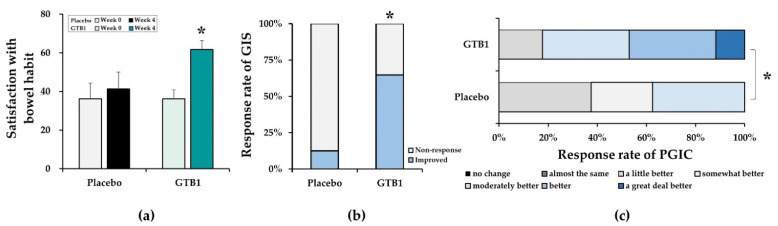
Bowel habit satisfaction assessment. (**a**) Changes in bowel habit satisfaction after GTB1 and placebo ingestion. (**b**) Global Improvement Scale (GIS) of IBS symptom ratings at end of intervention. GIS was evaluated on a seven-point scale, namely, improved (substantially improved or moderately improved), non-response (slightly improved, no change, slightly worse) and worse (substantially worse or moderately worse). (**c**) Impact of GTB1 and placebo ingestion on Patient’ Global Impression of Change (PGIC) at end of intervention. PGIC scores was recorded on a seven-point scale. Data are means ± SEM or % of participants. * *p* < 0.05 as determined by two-sample *t*-test or Chi-square test.

At baseline, the subjective bowel habit satisfaction scores were 36.25 ± 8.00 and 36.11 ± 4.65 for the placebo and GTB1 groups, respectively, and the difference was not significant. After four weeks ingestion, bowel habit satisfaction in the GTB1 group significantly improved by 25.56 ± 4.44 over baseline (*p* < 0.001). By contrast, no significant change in the placebo group was found over this time. Subjective bowel habit satisfaction significantly differed between the placebo and GTB1 groups by the end of ingestion (*p* = 0.041; Figure 6a). Global Improvement Scale (GIS) assessment of the IBS symptoms indicated that 64.7% of the participants in the GTB1 group had “improved” as they chose “substantially improved” or “moderately improved”. The remaining 36.3% chose “non-response”. For the placebo group, 12.5% chose “improved” while 87.5% chose “non-response”. There was a significant difference between the placebo and GTB1 groups (*p* = 0.015; Figure 6b). Impact on quality of life was measured using PGIS and was significantly improved in the GTB1 group compared to that in the placebo group (*p* = 0.013; Figure 6c).

### 3.6. Fecal Microbiota

**Figure 7:**
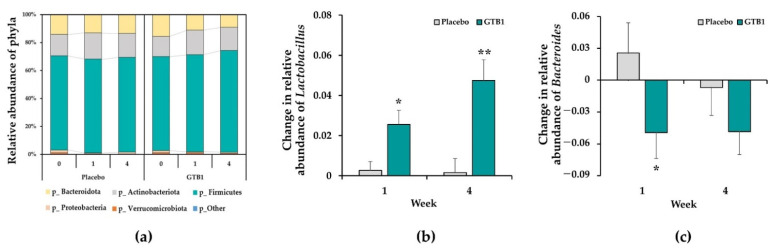
Fecal microbiota. (**a**) Proportions of various bacterial phyla comprising fecal microbiota. (**b**) Change in relative abundance of *Lactobacillus*. (**c**) Change in relative abundance of *Bacteroides*. Data are means ± SEM. * *p* < 0.05, ** *p* < 0.01 as determined by two-sample *t*-test.

Fecal microbial compositions were subjected to a taxon-dependent analysis. Firmicutes, Actinobacteriota, and Bacteroidetes were the dominant bacterial phyla followed by Proteobacteria, Verrucomicrobiota, and others (Figure 7a). At baseline, the abundances of Firmicutes, Actinobacteriota, Bacteroidetes, and Proteobacteria did not significantly differ between the GTB1 and placebo groups. For the GTB1 group, the amount of Firmicutes increased and Bacteroidetes decreased at one week after ingestion. By week four, the abundances of the foregoing bacterial phyla markedly differed from those of the placebo group. Compared to that in the placebo group, the relative abundance of *Lactobacillus* in the GTB1 group significantly increased from one week after ingestion (*p* = 0.046) and increased until week four (*p* = 0.010, Figure 7b). The relative abundance of *Bacteroides* in the GTB1 group significantly decreased after one week of ingestion compared to that in the placebo group (*p* = 0.034, Figure 7c). A significant correlation was observed when *Lactobacillus* genus abundance increased as the frequency of diarrhea (BSFS 6 to 7) decreased in the GTB1 group (*p* = 0.035, Figure S3a). In the GTB1 group, a correlation was observed when *Bacteroides* genus abundance decreased as the frequency of diarrhea decreased (Figure S3b).

## 4. Discussion

The present study aimed to assess the effectiveness of green tea-derived *Lactiplantibacillus plantarum* APsulloc 331261 (GTB1^TM^) at improving intestinal discomfort symptoms, frequency of defecation, and quality of life in IBS-D participants and to identify changes in fecal microbiota resulting from the GTB1 treatment. In the GTB1 group, intestinal discomfort symptoms such as abdominal pain, abdominal bloating, and feeling of incomplete evacuation were significantly improved compared those in with the placebo group. Frequency of diarrhea and number of bowel movements significantly decreased and bowel habit satisfaction improved in the GTB1 group relative to those in the placebo group. Improvement in IBS symptoms was also confirmed through subject GIS. PGIC and quality of life were significantly improved in the GTB1 group compared to those in the placebo group. Fecal microbiome analyses disclosed that GTB1 ingestion significantly increased the abundances of *Lactobacillus* and Firmicutes and decreased the abundance of *Bacteroides* and Bacteroidetes compared to the placebo group. There were no severe adverse events at any time during the study.

IBS involves several complex pathophysiological mechanisms such as intestinal barrier dysfunction, gut immune dysfunction, visceral hypersensitivity, gut microbiota dysbiosis, and dysfunctional gut-brain interactions [5,6,7,8,9,30]. Gut microbial dysbiosis causes the activation of the gut immune response, leading to epithelial barrier disorders [30]. The intestinal epithelial barrier is a primary line of defense against external stimuli and restricts the passage of harmful microorganisms and antigens [31]. Dysregulation of the intestinal epithelial barrier increases intestinal permeability, causes low-grade immune cell infiltration into the gut mucosa, and induces IBS-D [31,32]. Probiotics prevent pathogen adhesion to intestinal epithelial cells, suppress diarrhea-causing pathogenic microorganisms and intestinal harmful bacteria, and improve diarrhea symptoms [33,34]. They increase the effectiveness of the immunologic system and attenuate inflammation. [35]. In particular, IBS patients show an increase in pro-inflammatory cytokines such as tumor necrosis factor-α (TNF-α), interleukin (IL)-6, and interferon-γ (IFN-γ) [36,37]. *Lp. plantarum* 299v reduced IFN-γ levels in an animal model of IBS [38]. In an entero-toxigenic *Escherichia* (*E.*) *coli*-induced mouse diarrhea model, *Lp. plantarum* CCFM1143 down-regulated pro-inflammatory cytokines such as IFN-γ, TNF-α, and IL-6 [39]. A trial on chronic diarrhea in humans reported that modulation of intestinal inflammation via IL-6 inhibition improved the symptoms [20]. Exopolysaccharides from *Lp. plantarum* YW11 reduced pro-inflammatory cytokines TNF-α, IFN-γ, and IL-12 and increased the anti-inflammatory cytokine IL-10, improving the immune response [40]. GTB1 inhibited IL-6 and promoted the anti-inflammatory cytokine IL-10 in previous gastrointestinal studies [23]. In addition, probiotics not only inhibit inflammation of the intestinal epithelial barrier, but also promote mucin secretion to block pathogen adhesion [41]. *Lactobacillus* upregulated *MUC2* and/or *MUC3* in the epithelium of the small intestine, protected its mucosal layer, and weakened the adhesion of the entero-pathogen *E. coli* E2348/69 [42,43]. GTB1 more tightly adhered to Caco-2/TC-7 intestinal epithelial cells than *Lp. plantarum* ATCC 14917 or *Lp. plantarum* 299v [22]. The potent anti-inflammatory and intestinal adhesion efficacy of GTB1 should help maintain the integrity of the intestinal barrier and improve IBS symptoms.

IBS is defined as disorders of gut-brain interactions in Roman IV [7]. The gut-brain axis regulates gut function by coordinated communication between the gut and the brain, and helps indirect signal transmission between the host and gut microbiota [44]. Gut microbial dysbiosis degrades brain function by deregulating the production of gut microbiota metabolites such as short-chain fatty acids (SCFAs) and the synthesis or consumption of neurotransmitters such as 5-hydroxytryptophan (HT), which then induces gut dysmotility, visceral sensing, and visceral hypersensitivity [5,14]. Several studies have suggested that probiotics improve symptoms of diarrhea caused by gut microbial dysbiosis by regulating SCFAs production [39,45]. In an entero-toxigenic *E. coli*-induced mouse diarrhea model, *Lp. plantarum* CCFM1143 increased SCFAs production [39]. *Lp. plantarum* 299v has been shown to enhance the concentrations of fecal SCFAs such as butyrate in patients with recurrent *Clostridium.difficile*–associated diarrhea [45]. GTB1 increased SCFAs (acetate, propionate, and butyrate) in the *H. pylori*-infected mouse gastric mucosal erosion model [23]. As a result of these studies, the increase in SCFA may have affected the decrease in diarrhea in the GTB1 group. The gut dysmotility is associated with the changes in serotonin metabolism related to 5-HT [46]. Increased 5-HT levels in patients with IBS-D can lead to visceral hypersensitivity, and may act in concert with intensified sensory reactions and exacerbate pain perception [47,48,49]. IBS patients have reported that abdominal pain did not improve with defecation; rather, their condition worsened [8]. The present study showed a correlation between the number of abnormal bowel movements (BSFS type 6–7) and severity of abdominal pain at baseline. However, severity and frequency of abdominal pain, bloating, and feeling of incomplete evacuation, and the number of abnormal bowel movements decreased after ingestion of GTB1. In particular, the abdominal pain was confirmed to decrease with the number of abnormal bowel movements (BSFS type 6–7) after GTB1 ingestion. The feeling of incomplete evacuation was significantly correlated with a decrease in the frequency of diarrhea (data not shown). Ducrotté et al. [17] reported a decrease in abdominal pain, bloating, and the feeling of incomplete evacuation and frequency of defecation after four weeks of *Lp. plantarum* ingestion. Overall IBS symptoms improved in 95% of patients in the *Lp. plantarium* 299V group versus 15% of patients in the placebo group (*p* < 0.001). In IBS-D, defecation habits are unpredictable and more urgent than those of the other IBS subtypes. Hence, patients with IBS-D may have a poor quality of life because of numerous restrictions in diet and daily activity [50]. In terms of the overall quality of life assessment including limitations in daily activity, diet, and emotional distress caused by IBS symptoms, the GTB1 group presented with significant improvement compared to the placebo group. In the former group, 82.4% of all participants felt ‘moderately to a great deal better’ after the treatments. Therefore, the ingestion of *Lp. plantarum* improves abdominal discomfort symptoms, abnormal bowel habits, and quality of life in patients with IBS-D.

Many studies suggest that gut microbial dysbiosis induces changes in the gut microbiota composition, reducing the α-diversity of gut microbiota [5,9,51,52,53,54]. In particular, low microbial diversity in the small intestine increases permeability and causes diseases such as IBS-D [53,54]. A study of the difference between patients with IBS and healthy controls in terms of their intestinal microbial populations revealed that the former group presented with relatively low *Lactobacillus, Bifidobacterium,* and *Faecalibacterium* abundance and high *Enterobacteriaceae* and *Bacteroides* abundance [53,55]. GTB1 treatment increased the relative abundance of Firmicutes and decreased the relative abundance of Bacteroidetes. *Lp. plantarum* CCFM1143 intake increased Firmicutes and Proteobacteria abundance and decreased Bacteroidetes abundance [20]. The relative abundance of *Lactobacillus* and *Veillonella* (Phyla Firmicutes) was higher while that of *Escherichia–Shigella* (Phyla Proteobacteria) and *Bacteroides* (Phyla Bacteroidetes) was lower in the GTB1 group. *Lactobacillales* were significantly decreased in patients with IBS-D [55]. A reduction in lactic acid content may damage the intestinal barrier, increase osmotic pressure in the intestinal lumen, and cause diarrhea [56]. *Lp. plantarum* and *L. acidophilus* inhibit *E. coli* growth [57]. Certain *Lactobacillus* produce the peptide bacteriocin which prevents the proliferation of various microbial pathogens [58]. *Veillonella* produces propionate [59] and its abundance was relatively higher in the GTB1 group. *Escherichia-Shigella* (Enterobacteriaceae) cause gastrointestinal disorders, constipation, or diarrhea and are implicated in post-infection IBS [60,61,62]. Their relative abundance was lower in the GTB1 group. *Bacteroides* cause diarrhea and occur mainly in patients with IBS-D. This may be because some species, such as toxigenic strains of *Bacteroides fragilis*, can promote the release of inflammatory factors from epithelial cells and thus cause inflammation [63], interfere with cell proliferation, damage DNA and promote the release of inflammatory factor from epithelial cells. However, GTB1 ingestion significantly reduced *Bacteroides* abundance [64]. After GTB1 ingestion, the Firmicutes/Bacteroidetes (F/B) ratio increased. This ratio can be used to predict health or disease, although age and existing pathological conditions must also be considered [65]. Disease risk increases with decreasing F/B ratio. For example, F/B ratio is low in patients with intestinal disorders such as inflammatory bowel disease [66,67]. However, GTB1 ingestion could increase the F/B ratio and help improve gut microbiota diversity. The preceding results suggested that changes to the gut microbiota made by GTB1 could be associated with symptomatic improvement in IBS-D. *Lp. plantarum* 299v administration significantly improved gut microbiota diversity and composition [68]. These clinical studies demonstrated that *Lactobacillus* could be a useful alternative to conventional IBS treatment. Nevertheless, other trials reported contradictory results; therefore, the efficacy of this treatment may vary in a strain-dependent manner.

The present study had certain limitations. Sample size was calculated based on prior studies. However, interpretation of the results was constrained by the fact that there were only 27 participants. Moreover, although treatment efficacy within the intervention period (four weeks) was confirmed, it is necessary to confirm the effect of long-term intake of GTB1 with a focus on the symptoms of IBS that recur frequently. The IBS-D subtype is strongly affected by diet. Therefore, patients were advised to maintain normal eating habits throughout the intervention period. However, no nutritional assessment was conducted of patients to confirm that they were following this recommendation. The effect of GTB1 was tested using only a single dose, namely, 10^10^ CFU/d. A systematic review of effects of probiotic intake in patients with IBS demonstrated improvement in global symptoms even at low doses (10^9^–10^10^ CFU/d) of *Lactobacillus* and *Bifidobacterium* [24]. Staudacher et al. [69] showed that taking more than 10^10^ CFU/d active probiotics may cause excessive carbohydrate fermentation and gas production and uncomfortable bowel habits. The Ministry of Food and Drug Safety of Korea proposed that 10^10^ CFU/d probiotic intake is adequate. For these reasons, a dosage of 10^10^ CFU/d was adopted in the present study. Despite the foregoing limitations, the present study was the first successful double-blind, randomized, controlled clinical study of GTB1 derived from green tea and it successfully demonstrated that GTB1 ingestion realizes positive changes in the fecal microbiota of patients with IBS-D. The present work also revealed a correlation between significant increase in *Lactobacillus* abundance and improvement in IBS-D symptoms. Both the present and past studies indicated the beneficial effects of *Lactobacillus plantarum* ingestion in patients with IBS-D [70]. The results of the study revealed that GTB1 was efficacious as a probiotic for the improvement of symptoms in patients with IBS-D. Based on the results of the present work, the efficacy of GTB1 ingestion on other IBS subtypes will be investigated in future research.

## 5. Conclusions

GTB1 ingestion demonstrated greater clinical efficacy than the placebo at managing IBS-D. GTB1 ameliorates intestinal health in IBS-D by increasing the abundance of beneficial bacteria such as *Lactobacillus*, reducing intestinal discomfort, maintaining stool consistency, and improving quality of life. The results of this study could lay the foundation for the development of functional probiotics that can be administered to treat bowel diseases such as IBS-D with complex etiology and recurring symptoms.

## Figures and Tables

**Table 1:** Baseline demographics and clinical characteristics.

		Placebo (*n* = 8)	GTB1 (*n* = 18)	*p*-Value ^1^
		Mean (SD) or *n* (%)	Mean (SD) or *n* (%)	
Age (years)		39.38 (3.16)	39.84 (6.44)	0.848
Female gender, ***n***(%)		6 (75.00%)	12 (66.67%)	0.568
BMI (kg/m^2^)		24.40 (3.84)	24.00 (3.44)	0.797
Systolic blood pressure (mmHg)		108.88 (7.16)	110.94 (7.63)	0.522
Diastolic blood pressure (mmHg)		81.13 (5.74)	79.28 (6.00)	0.470
Rome IV	Related to defecation	7 (87.50%)	16 (84.21%)	0.834
	Change in frequency of stool	6 (75.00%)	14 (73.68%)	0.946
	Change in form of stool	8 (100.00%)	18 (94.74%)	0.527
Intestinal discomfort	Abdominal pain	5.25 (1.39)	5.83 (1.58)	0.378
	Abdominal bloating	4.75 (3.11)	5.50 (2.87)	0.571
	Feeling of incomplete evacuation	4.00 (2.88)	6.17 (2.62)	0.093
Defecation condition	Defecation frequency(/week)	9.95 (2.56)	10.71 (2.64)	0.504
	BSFS types 6 & 7 (%)	63.22 (21.40)	69.07 (19.14)	0.494

^1^ Between-group comparisons using a two-sample *t*-test or Wilcoxon rank-sum test for continuous variables and Chi-square test for categorical variables. BMI: Body Mass Index; BSFS: Bristol stool form scale.

**Table 2:** Comparison of intestinal discomfort symptoms during study periods.

	Abdominal Pain		Abdominal Bloating		Feeling of Incomplete Evacuation	
	Placebo	GTB1	Placebo	GTB1	Placebo	GTB1
Week 0	5.25 ± 1.39	5.83 ± 1.58	4.75 ± 3.11	5.50 ± 2.87	4.00 ± 2.88	6.17 ± 2.62
Week 1	3.63 ± 2.45	3.83 ± 2.73 ^##^	4.63 ± 2.50	3.61 ± 2.52 ^#^	3.75 ± 1.91	4.39 ± 2.43 ^#^
Week 2	3.75 ± 1.98	2.67 ± 1.75 ^###^	3.75 ± 1.98	2.83 ± 2.12 ^##^	3.38 ± 2.39	3.67 ± 2.63 ^##^
Week 4	3.75 ± 2.60	1.67 ± 1.08 ^###,^**	3.63 ± 3.07	2.11 ± 1.49 ^###,^*	3.88 ± 2.30	2.89 ± 2.27 ^##,^*
Week 4 + 2	3.63 ± 2.39	2.33 ± 1.81 ^###^	4.38 ± 3.07	2.89 ± 2.05 ^###,^*	4.50 ± 2.56	2.78 ± 2.34 ^###,^***

Data are means ± SD. ^#^, *p* < 0.05; ^##^, *p* < 0.01; ^###^, *p* < 0.001; Differences from baseline within groups were determined by paired *t*-test or Wilcoxon signed-rank test. *, *p* < 0.05; **, *p* < 0.01; ***, *p* < 0.001 compared with the placebo group according to two-sample *t*-test or Wilcoxon rank-sum test.

## Data Availability

Data sharing is not applicable to this article due to ethical policy.

## Supplementary Materials

The following supporting information can be downloaded at: https://www.mdpi.com/article/10.3390/nu14102015/s1, Figure S1: Improvement rate (%) of intestinal discomfort. Figure S2: Stool consistency; Figure S3: Correlation between abnormal defecation frequency and fecal microbiota.
