# Supplementary material for: Effect of Oral Intake of Lactiplantibacillus plantarum APsulloc 331261 (GTB1TM) on Diarrhea-Predominant Irritable Bowel Syndrome: A Randomized, Double-Blind, Placebo-Controlled Study
Source: Nutrients. 2022 May 11;14(10):2015. doi: 10.3390/nu14102015 (PMC9144213; doi:10.3390/nu14102015)
Supplement: Supplementary file 1 [file nutrients-14-02015-s001.zip › nutrients-1697937-supplementary.pdf]

nutrients MDPI

Supplementary Materials:

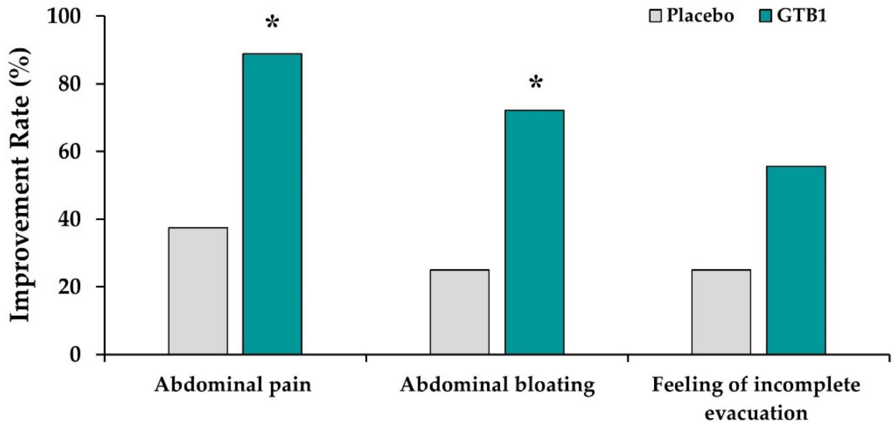

Figure S1. Improvement rate (%) of intestinal discomfort. Percentage of participants who decreased by 50% compared to the baseline in 4-week after ingestion. * $p < 0.05$ as determined by Chi-square test.

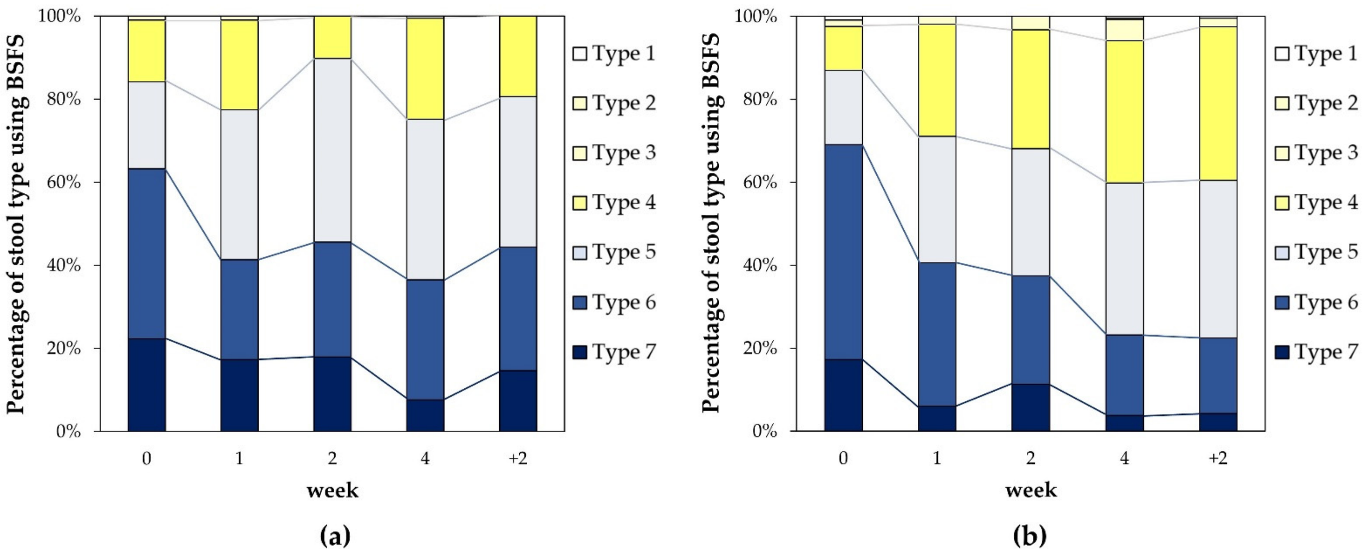

Figure S2. Stool consistency. (**a**) Placebo group. (**b**) GTB1 group. The stool record according to Bristol stool form scale is recorded for seven days before each visit during the baseline (Week 0), intake period (Week 1, 2, and 4), and follow-up period (two weeks after ingestion). It shows the rate of change of stool type according to BSFS for each visit.

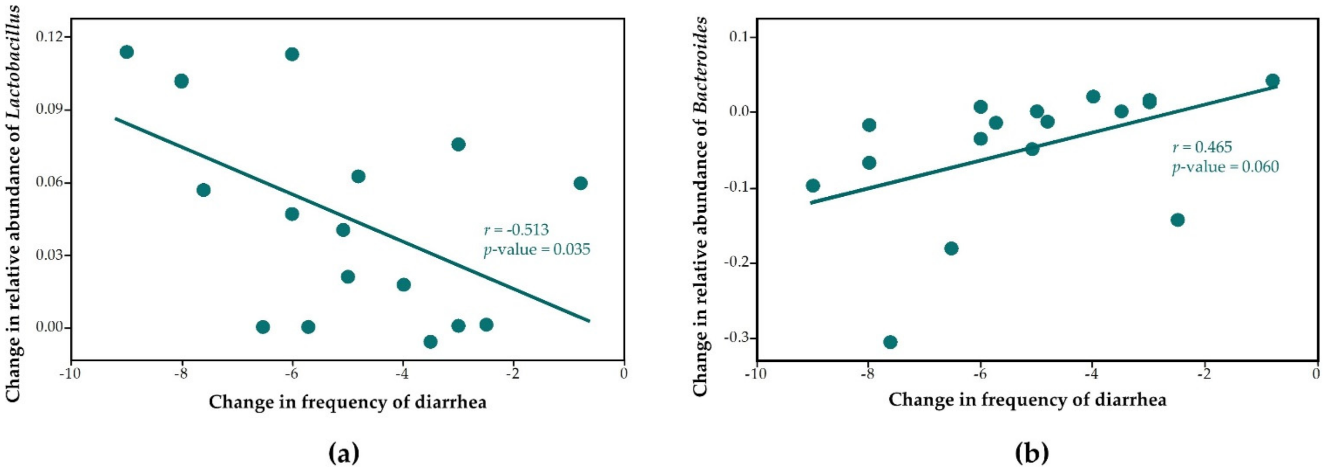

Figure S3. Correlation between abnormal defecation frequency and fecal microbiota. (**a**) Correlation between changes in the frequency of diarrhea (BSFS type 6–7) and *Lactobacillus* abundance over four weeks. (**b**) Correlation between changes in the frequency of diarrhea and *Bacteroides* abundance. Dot indicates individual participants in GTB1 (●) group. Lines indicate optimal linear fit through all data points. R value is Pearson's correlation coefficient.
